# Reexamining the Health Implications of Objective and Subjective Neighborhood Measures: New Insights from REWARD

**DOI:** 10.1093/geroni/igaf122.2725

**Published:** 2025-12-31

**Authors:** Meiyi Li, Wei Xu, Christina Kamis, Amy Schultz, Kristen Malecki, Michal Engelman

**Affiliations:** University of Wisconsin–Madison, Madison, Wisconsin, United States; Medical College of Wisconsin, Milwaukee, Wisconsin, United States; University of Illinois Urbana-Champaign, Champaign, Illinois, United States; University of Wisconsin–Madison, Madison, Wisconsin, United States; University of Illinois Chicago, Chicago, Illinois, United States; University of Wisconsin-Madison, Madison, Wisconsin, United States

## Abstract

Neighborhood is recognized as the key determinant of individual well-being and population health. Despite evidence on neighborhood and health, how neighborhoods should be measured remains contested. The scholarship has continuously debated over objective versus subjective measures of neighborhoods and their relative importance on health. Further, the process by which neighborhoods “get under the skin” to affect health remains to be examined. The current study draws upon longitudinal residential history and novel epigenetic biomarkers of aging from the Researching Epigenetics, Weathering, Aging, & Disadvantage Study (REWARD) to provide new insights into this ongoing debate. We aim to investigate (a) the relationship between objective neighborhood disadvantages and subjective perceptions of living in the neighborhoods and accelerated biological aging, (b) the relative importance of objective and subjective neighborhood measures, and (c) the extent to which subjective perception moderates the association between objective neighborhood disadvantages and accelerated biological aging. Preliminary results demonstrate that both objective neighborhood disadvantages and subjective perceptions are associated with accelerated biological measures, with objective neighborhood disadvantages showing stronger impacts. Further, the relationship between objective neighborhood disadvantages and accelerated biological aging cannot be moderated by subjective perceptions. Results from this study not only contribute to the ongoing debate of neighborhood measures but also shed light on the underlying pathway by which neighborhoods “get under the skin” to affect aging and health.

